# Supplementary material for: Patient-specific responses to SMN2 splice-modifying treatments in spinal muscular atrophy fibroblasts
Source: Mol Ther Methods Clin Dev. 2024 Nov 13;32(4):101379. doi: 10.1016/j.omtm.2024.101379 (PMC11626024; doi:10.1016/j.omtm.2024.101379)
Supplement: Document S1. Figures S1–S8 and Tables S1–S3 [file mmc1.pdf]

OMTM, Volume *32*

Supplemental information

Patient-specific responses to *SMN2* splice-modifying treatments in spinal muscular atrophy fibroblasts

Ilaria Signoria, Maria M. Zwartkruis, Lotte Geerlofs, Elena Perenthaler, Kiterie M.E. Faller, Rachel James, Harriet McHale-Owen, Jared W. Green, Joris Kortooms, Sophie H. Snellen, Fay-Lynn Asselman, Thomas H. Gillingwater, Gabriella Viero, Renske I. Wadman, W. Ludo van der Pol, and Ewout J.N. Groen

Table S1. **Overview of primer and probe sequences used for ddPCR analyses.**

Target	Primer/Prob	Sequence
SMN1	FW	TAC ATG AGT GGC TAT CAT ACT GGC TA
	Probe	5'-6FAM TAT GGG TTT CAG ACA AA MGB-3'
	RV	AAT GTG AGC ACC TTC CTT CTT TTT
SMN2	FW	AC ATG AGT GGC TAT CAT ACT GGC TA
	Probe	5'-6FAM ATA TGG GTT TTA GAC AAA A MGB-3'
	RV	AAT GTG AGC ACC TTC CTT CTT TTT
SMN∆7	FW	GG CTA TCA TAC TGG CTA TTA TAT GGA A
	Probe	5'-6FAM CTG GCA TAG AGC AGC ACT AAA TGA CAC CAC MGB-3'
	RV	TCC AGA TCT GTC TGA TCG TTT CTT
TBP	FW	CGT GGT TCG TGG CTC TCT
	Probe	5’-HEX ATC CCA AGC -ZEN- GGT TTG CTG-3’
	RV	GCC CGA AAC GCC GAA TAT

Target name, primer or probe and sequences are indicated. FW = forward; RV = reverse. The probes are FAM-MGB (*SMN*) or HEX-ZEN (*TBP*). For references to previous studies using these sequences, please see the main text of our manuscript (**Materials and methods**).

Table S2. Overview of SMA and control clinical and genetic characteristics.

		Type 1b (n=4)	Type 1c (n=5)	Type 2 (n=14)	Type 3 (n=8)	Type 4 (n=4)	Control (n=10)
Sex (M:F)		2:2	3:2	9:5	5:3	3:1	4:6
SMN1 CN	0	4	5	14	8	4	0
	1	0	0	0	0	0	0
	2	0	0	0	0	0	8
	3	0	0	0	0	0	1
	4	0	0	0	0	0	1
SMN2 CN	1	0	0	0	0	0	5
	2	4	0	0	0	0	5
	3	0	5	14	4	0	0
	4	0	0	0	4	4	0
Median age in months at onset (range)		1.8 (0-2.5)	6 (6-9)	12 (6-36)	39 (12-168)	327 (246-510)	-
Median age in years at biopsy (range)		0.8 (0-2)	27 (5-45)	7 (2-32)	20.5 (9-63)	49 (34-70)	28 (25-62)

Table S3. Estimates of the factor significant in the linear regression model for SMN levels (total amount and increase) after treatment.

**SMN after treatment**	Total amount		Increase	
Linear regression model variable	**Nusinersen** Estimate	**Risdiplam** Estimate	**Nusinersen** Estimate	**Risdiplam** Estimate
Intercept	0.15296	0.17158	0.15297	0.17158
SMN protein levels prior to treatment	1.45840	1.50690	0.45840	0.50689
SMN2Δ7 mRNA levels prior to treatment	-0.10463	-0.13511	-0.10463	-0.13511
Age group (pediatric/adult)	-0.06415	-0.05990	-0.06415	-0.05990
SMN2 copy number (3x *SMN2*)	0.09871	0.11985	0.09871	0.11985
SMN2 copy number (4x *SMN2*)	0.13355	0.17212	0.13355	0.17212

A

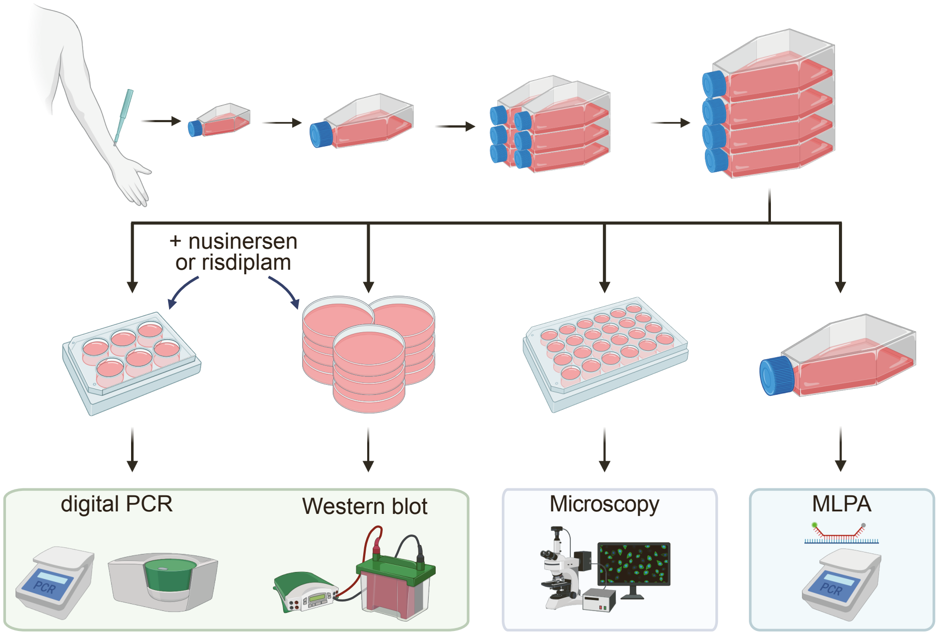

Figure S1. Workflow for the rapid expansion and culturing of primary-derived fibroblasts to obtain sufficient, low-passage numbers of cells as used in this study. Figure made with Biorender.com.

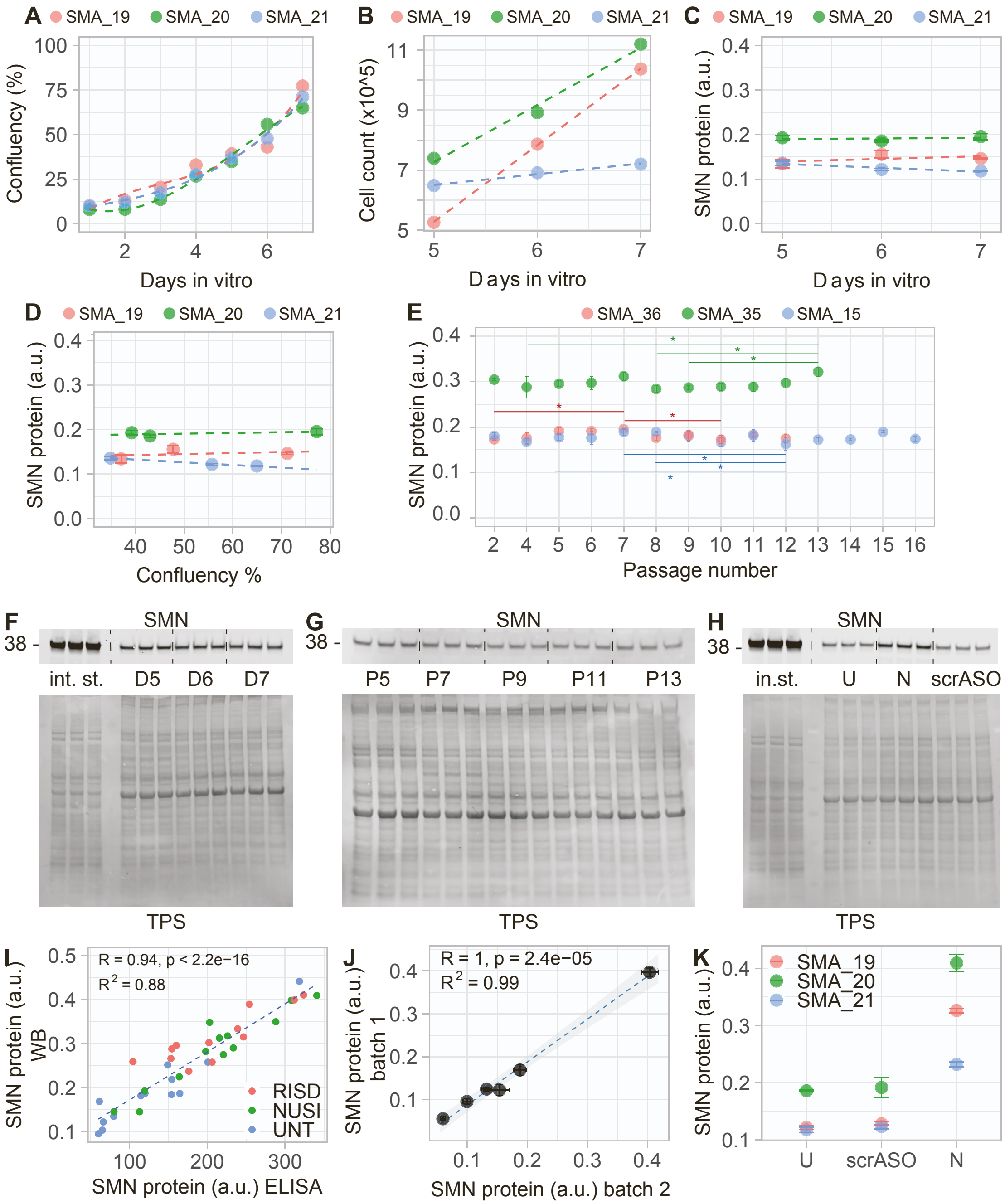

Figure S2. Control analyses for SMN protein measurements in primary fibroblasts.

(A) Cell confluency measurement after 1 to 7 days *in vitro* of three different SMA patient-derived primary fibroblast lines. Confluency increases consistently over time in different cell lines. **(B)** Cell count over a 10cm dish after 5, 6 and 7 days *in vitro* of three different SMA patient-derived primary fibroblast lines. Cell number increase is variable, although within the same order of magnitude. Data are represented as the

average of three biological triplicates. **(C)** Normalized SMN protein expression in SMA patient-derived primary fibroblast lines after 5, 6 and 7 days in culture. We found no statistically significant difference (*P* = 0.43, two-way ANOVA). Data are represented as the average of technical triplicates ± standard deviation. **(D)** Relationship between normalized SMN protein expression in SMA patient-derived primary fibroblasts and cell confluency. SMN protein expression does not depend on confluency (*P* = 0.41, two-way ANOVA). Data are represented as the average of technical triplicates ± standard deviation. **(E)** Normalized SMN protein expression in SMA patient-derived primary fibroblasts at different passage numbers. We observed some variability but did not identify any consistent changes in SMN protein levels over time for the passage numbers we investigated (P2 to P16). Data are represented as the average of technical triplicates ± standard deviation. (Tukey post-hoc test: SMA_36 P7 vs. P2 *P* = 0.03, P10 vs. P7 *P* = 0.03; SMA_35 P12 vs. P7 *P* = 0.04, P12 vs. P8 *P* =0.04, P12 vs. P15 *P* = 0.04; SMA_15 P13 vs. P4 *P* = 0.05, P13 vs. P9 *P* = 0.04, P13 vs. P8 *P* = 0.02). **(F)** Representative western blot of SMN protein expression in SMA patient-derived primary fibroblasts at different days in vitro (D5, D6, D7) as used for analyses in (**C**) and (**D**). **(G)** Representative western blot of SMN protein expression in SMA patient-derived primary fibroblasts at different passage numbers (P5, P7, P9, P11, P13). **(H)** Representative western blot of SMN protein expression in SMA patient-derived primary fibroblast cell line untreated (U) and after *in vitro* treatment with either nusinersen (N) or a scrASO (scrASO). **(I)** Correlation between normalized SMN protein quantification via semi-quantitative western blotting (WB) and ELISA of 38 different samples untreated or treated with nusinersen or risdiplam (P < 2.2e-16, R^2 = 0.88). Data are represented as the average of the technical triplicate ± standard deviation. The regression line (dashed line), its 95% confidence level interval, Pearson correlation coefficient (R), *P* value (p) and the coefficient of determination (R^2) are displayed. **(J)** Correlation between SMN expression in two batches of the same cell lines thawed at two distinct time points. There is a strong and significant correlation (*P* = 2.4e-05, R^2 = 0.99). Data are represented as the average of the technical triplicate ± standard deviation. The regression line (dashed line), its 95% confidence level interval, Pearson correlation coefficient (R), *P*-value (p) and the coefficient of determination (R^2) are displayed. **(K)** SMN protein expression levels in three SMA patient-derived cell lines untreated (U), treated *in vitro* with either a scrASO or nusinersen (N) through lipofection. A representative western blot is shown in panel **H**. Data are represented as the average of the technical triplicate ± standard deviation. TPS = total protein

staining; in. st = internal standard; N = nusinersen.; scrASO = scrambles antisense oligonucleotide; a.u. = arbitrary unit; * = $P < 0.05$.

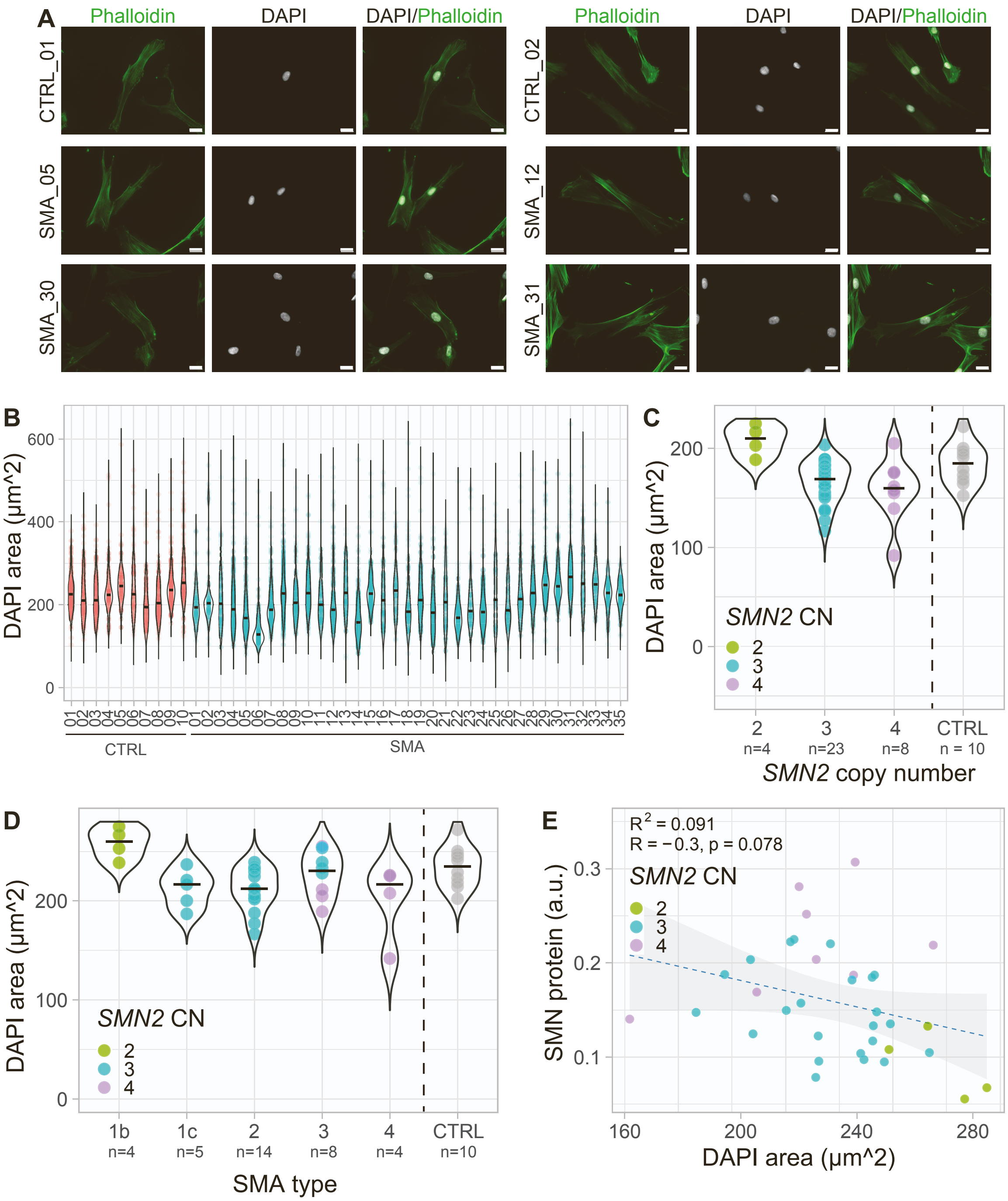

Figure S3. Morphological characterization of control and SMA patient-derived fibroblasts. (A) Representative images for control and SMA patient-derived fibroblasts. F-actin was labelled using phalloidin conjugated to Alexa Fluor 488. Chromatin was labelled using DAPI. SMA_05 and SMA_12 respectively have 4x *SMN2* and 3x *SMN2* copies. SMA_30 and SMA_31 have 2x *SMN2*. Scale bar: 25 µm. **(B)** DAPI (nucleus) area (µm²) in control and SMA patient-derived primary fibroblasts. Each dot corresponds

to the area of one nucleus. **(C)** Average DAPI area (µm²) of control (n=10) and SMA patient-derived primary fibroblasts with 2x *SMN2* copies (n=4), 3x *SMN2* copies (n=23) and 4x *SMN2* copies (n=8). Nuclear size depends on *SMN2* copy number (one-way ANOVA, *P*=0.002, 2 vs. 3 p=0.009; 2 vs. 4 *P*=0.005). Each dot corresponds to the average nuclear size of each cell line. **(D)** DAPI area (µm²) of control (n=10) and SMA type 1b (n=4), type 1c (n=5), type 2 (n=14), type 3 (n=8) and type 4 (n=4) patient-derived primary fibroblasts. Nuclear size is partially related to SMA type (one-way ANOVA, *P*= 0.004, 1b vs. 1c *P*= 0.05; 1b vs. 2 *P*= 0.01; 1b vs. 4 p=0.01). Each dot corresponds to the average nuclear size of each cell line. **(E)** Relationship between normalized SMN protein expression levels (a.u.) and DAPI area (µm²) in SMA patient-derived primary fibroblasts. SMN protein does not correlate with nuclear area (*P* = 0.08). Data are represented as the average nuclear area of each cell line. The regression line (dashed line), its 95% confidence level interval, Pearson correlation coefficient (R), *P*-value (p) and the coefficient of determination (R^2) are displayed. CN = copy number.

.

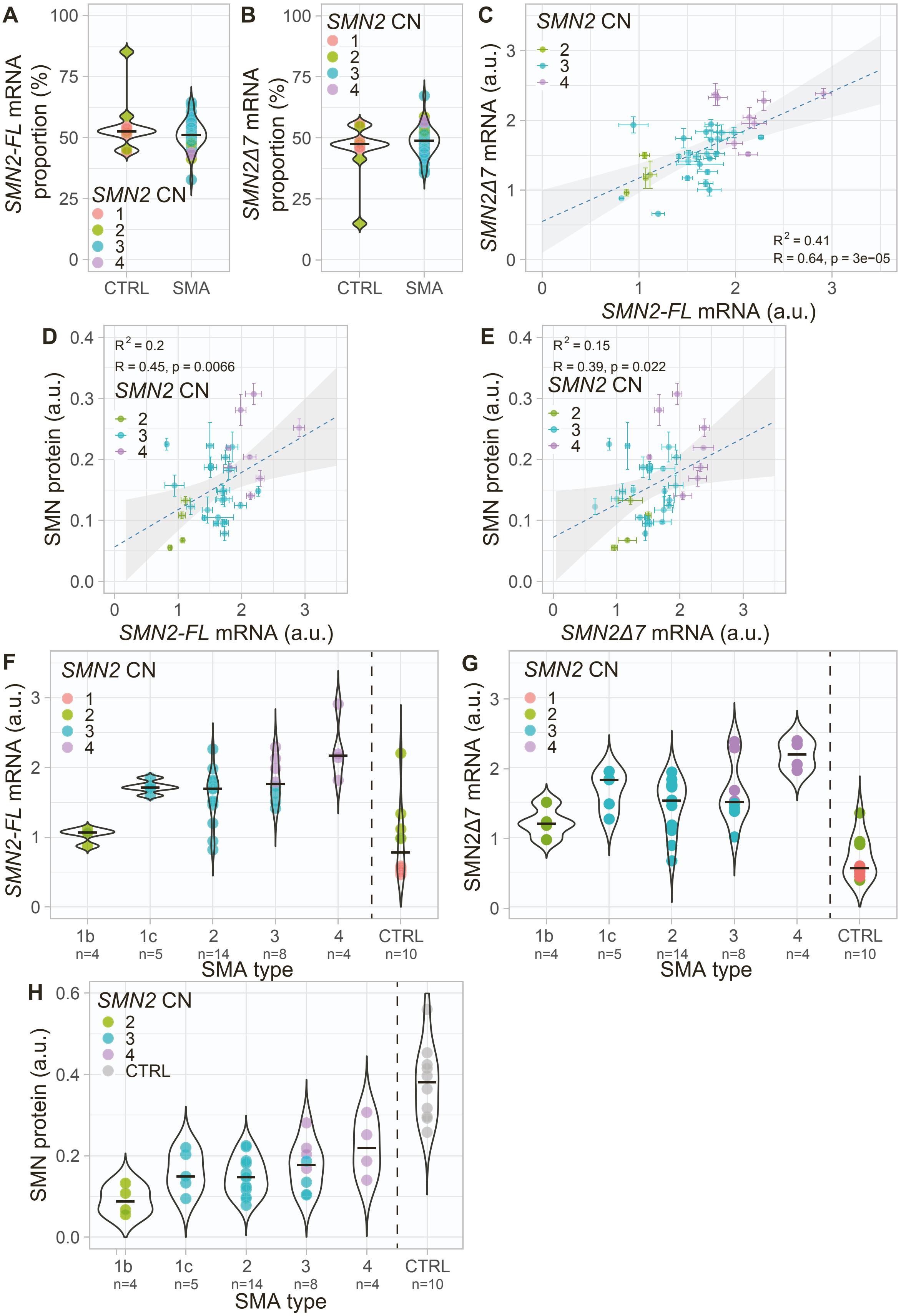
A
SMN2-FL mRNA proportion (%)
100
75
50
25
0
CTRL
SMA
SMN2 CN
1
2
3
4
B
SMN2Δ7 mRNA proportion (%)
C
SMN2Δ7 mRNA (a.u.)
SMN2-FL mRNA (a.u.)
R2 = 0.41
R = 0.64, p = 3e−05
D
SMN protein (a.u.)
R2 = 0.2
R = 0.45, p = 0.0066
E
R2 = 0.15
R = 0.39, p = 0.022
SMN2Δ7 mRNA (a.u.)
F
SMN2-FL mRNA (a.u.)
1b
n=4
1c
n=5
2
n=14
3
n=8
4
n=4
CTRL
n=10
SMA type
G
SMN2Δ7 mRNA (a.u.)
H
SMN protein (a.u.)
CTRL

Figure S4. SMN protein and *SMN2* mRNA correlation with clinical features in patients-derived primary fibroblasts. (A) Percentage of *SMN2-FL* mRNA relative to total *SMN2* mRNA in control (n=10) and SMA patient-derived primary fibroblasts (n=35). There is no statistically significant difference in the relative abundance of *SMN2-FL* in control and SMA patient-derived fibroblasts. Each dot corresponds to the average of the technical triplicate of each cell line. **(B)** Percentage of *SMN2Δ7* mRNA relative to total *SMN2* mRNA in control (n=10) and SMA patient-derived primary fibroblasts (n=35). There is no statistically significant difference in the relative abundance of *SMN2Δ7* in control and patient-derived fibroblasts. Each dot corresponds to the average of technical triplicate of each cell line. **(C)** Relationship between *SMN2-FL* and *SMNΔ7* mRNA expression levels in SMA patient-derived primary fibroblasts. *SMN2-FL* and *SMN2Δ7* mRNA expression significantly correlates (P = 3e-05, R^2 = 0.41). Data are represented as the average of the technical triplicate ± standard deviation. The regression line (dashed line), its 95% confidence level interval, Pearson correlation coefficient (R), P-value (p) and the coefficient of determination (R^2) are displayed. **(D)** Relationship between *SMN2-FL* mRNA and normalized SMN protein expression levels in SMA patient-derived primary fibroblasts. *SMN2-FL* mRNA significantly correlates with SMN protein levels (P = 0.007). Data are represented as the average of the technical triplicate ± standard deviation**.** The regression line (dashed line), its 95% confidence level interval, Pearson correlation coefficient (R), P-value (p) and the coefficient of determination (R^2) are displayed. **(E)** Relationship between *SMN2Δ7* mRNA and normalized SMN protein expression levels in SMA patient-derived primary fibroblasts. *SMN2Δ7* mRNA correlates with SMN protein production (P = 0.02). Data are represented as the average of the technical triplicate ± standard deviation. The regression line (dashed line), its 95% confidence level interval, Pearson correlation coefficient (R), P-value (p) and the coefficient of determination (R^2) are displayed. **(F)** *SMN2-FL* mRNA expression in control (n=10) and SMA type 1b (n=4), type 1c (n=5), type 2 (n=14), type 3 (n=8) and type 4 (n=4) patient-derived primary fibroblasts. There are differences in *SMN2-FL* mRNA expression across different SMA types (one-way ANOVA P = 0.0003, 1b vs. 1c P = 0.03, 1b vs. 2 P = 0.045, 1b vs. 3 P = 0.005, 1b vs. 4 P = 0.0001, 2 vs. 4 P = 0.00). Each dot corresponds to the average of technical triplicate of each cell line. **(G)** *SMN2Δ7* expression in control (n=10) and SMA type 1b (n=4), type 1c (n=5), type 2 (n=14), type 3 (n=8) and type 4 (n=4) patient-derived primary fibroblasts. There are some differences in *SMN2Δ7* mRNA expression across different SMA types (one-way ANOVA P 0.009, 1b vs. 4 P = 0.006, 2

vs. 4 *P* = 0.01). Each dot corresponds to the average of technical triplicate of each cell line. **(H)** Normalized SMN protein expression levels in control (n=10) and SMA type 1b (n=4), type 1c (n=5), type 2 (n=14), type 3 (n=8) and type 4 (n=4) patient-derived primary fibroblasts. SMN protein expression does not depend on SMA type (one-way ANOVA, *P* = 0.02, 1b vs. 4 *P* = 0.01). Each dot corresponds to the average of a technical triplicate for each cell line. a.u. = arbitry unit; CN = copy number.

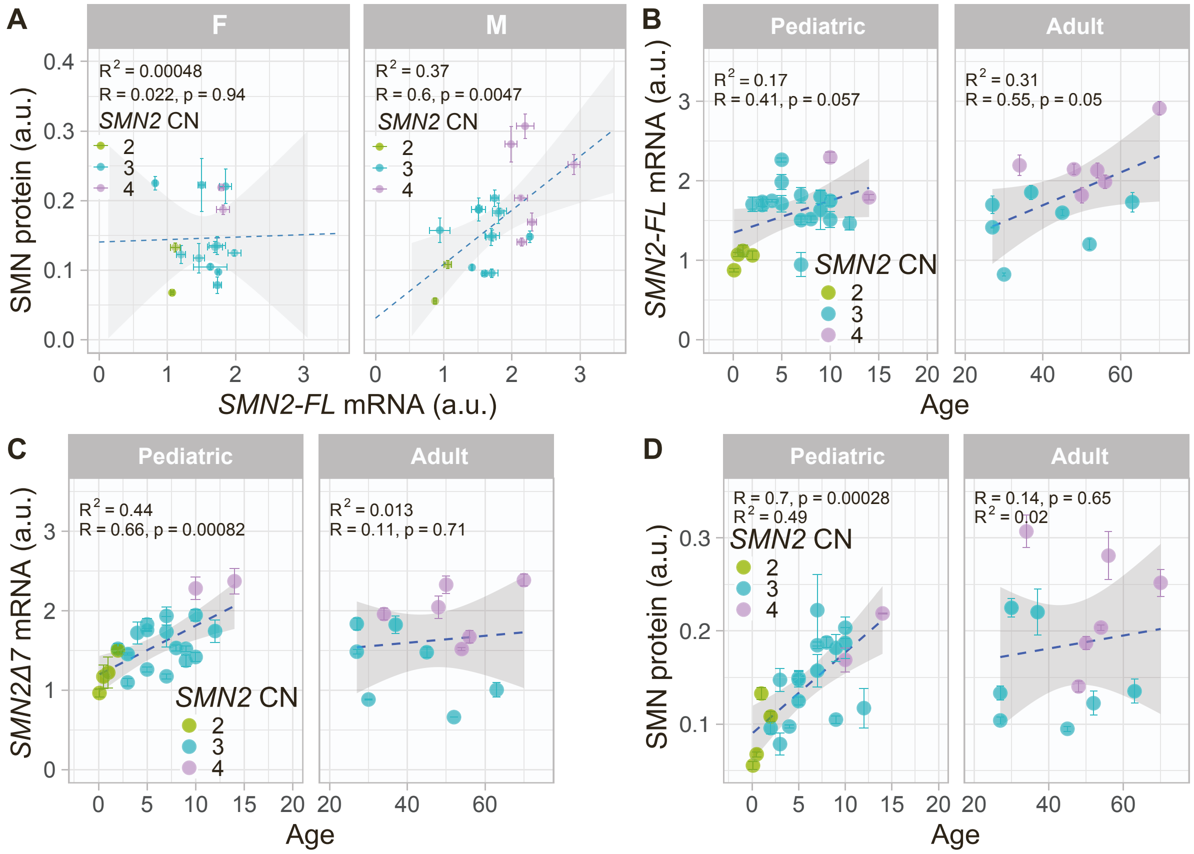

Figure S5. SMN protein and *SMN2* mRNA correlation with age and sex in patients-derived primary fibroblasts. (A) Relationship between *SMN2-FL* mRNA and normalized SMN protein expression levels in SMA patient-derived primary fibroblasts from female and male donors. *SMN2-FL* mRNA and SMN protein correlation is influenced by the donors' sex. Data are represented as the average of the technical triplicate ± standard deviation. The regression line (dashed line), its 95% confidence level interval, Pearson correlation coefficient (R), p-value (p) and the coefficient of determination (R^2) are displayed. **(B)** Relationship between *SMN2-FL* mRNA expression and age at biopsy in SMA patient-derived primary fibroblasts. *SMN2-FL* mRNA expression shows trend of weak correlation with age in pediatric and adults (P = 0.057 and P = 0.05, respectively). Data are represented as the average of the technical triplicate ± standard deviation. Regression line (dashed line), Pearson correlation coefficient (R), p-value (p) and the coefficient of determination (R^2) are displayed. **(C)** Relationship between *SMN2Δ7* mRNA expression and age at the biopsy in SMA patient-derived primary fibroblasts. *SMN2Δ7* mRNA expression correlates with

age in pediatric (*P* = 0.0008) but not adult patients (*P* = 0.71). Data are represented as the average of the technical triplicate ± standard deviation. The regression line (dashed line), Pearson correlation coefficient (R), *P*-value (p) and the coefficient of determination (R^2) are displayed. **(D)** Correlation between normalized SMN protein expression and age at biopsy in SMA fibroblasts. Protein expression correlates with age in pediatric (*P* = 0.003) but not adult patients (*P* = 0.65). Data are represented as the average of the technical triplicate ± standard deviation. The regression line (dashed line), its 95% confidence level interval, Pearson correlation coefficient (R), p-value (p) and the coefficient of determination (R^2) are displayed. a.u. = arbitrary unit; CN = copy number.

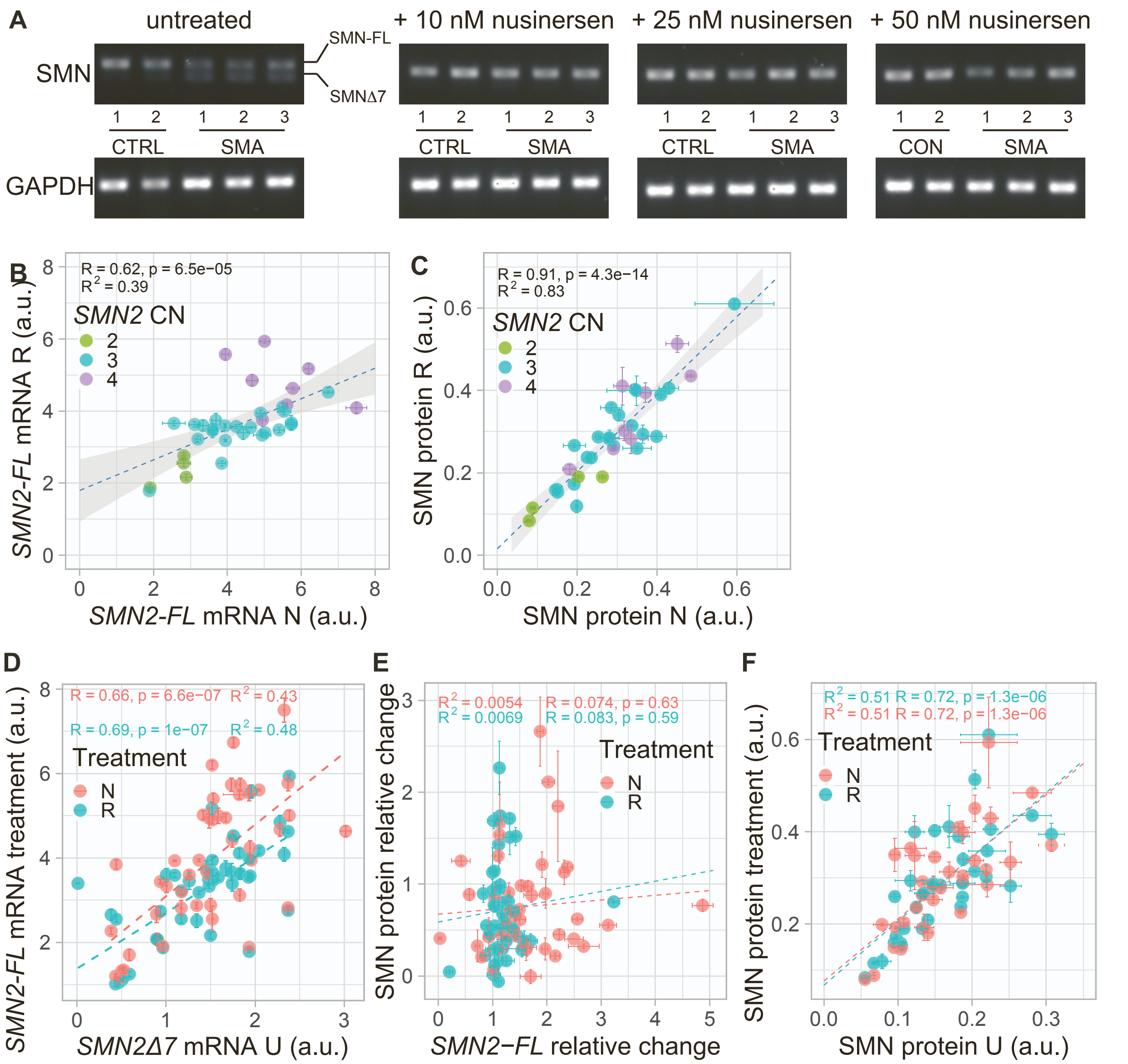

Figure S6. The effect of *SMN2*-splice modifiers on *SMN2-FL*, *SMN2*Δ*7* and SMN expression levels in control and SMA patient-derived primary fibroblasts.

(A) Agarose gel image of RT-PCR showing *SMN2* exon 7 splicing in control and SMA patient fibroblasts treated with nusinersen at different concentrations (10, 25 and 50 nM). **(B)** Relationship between *SMN2-FL* mRNA in SMA patient-derived primary fibroblasts treated with nusinersen (N) and risdiplam (R). Data are represented as the average of the technical triplicate ± standard deviation. The regression line, its 95% confidence level interval, Pearson correlation coefficient (R), p-value (p) and the coefficient of determination

(R^2) are displayed. **(C)** Relationship between normalized SMN protein in SMA patient-derived primary fibroblasts treated with nusinersen (N) and risdiplam (R). Data are represented as the average of the technical triplicate ± standard deviation. Regression line, its 95% confidence level interval, Pearson correlation coefficient (R), *P*-value (p) and the coefficient of determination (R^2) are displayed. **(D)** Correlation between *SMN2-FL* mRNA after *in vitro* treatment with either nusinersen (N) and risdiplam (R) and *SMNΔ7* mRNA level in untreated fibroblasts (U) in SMA patient-derived fibroblasts. There is a statistically significant correlation. Data are represented as the average of the technical triplicate ± standard deviation. The regression line, Pearson correlation coefficient (R), *P*-value (p) and the coefficient of determination (R^2) are displayed. **(E)** Relationship between *SMN2-FL* mRNA increase after *in vitro* treatment displayed as relative change and SMN protein increase after treatment displayed as relative change after treatment with either nusinersen (N, coral) and risdiplam (R, light blue) in SMA patient-derived primary fibroblasts. The correlation is not statistically significant for both treatments. Data are represented as the average of the technical triplicate ± standard deviation. Regression line, Pearson correlation coefficient (R), *P*-value (p) and the coefficient of determination (R^2) are displayed. **(F)** Relationship between normalized SMN protein in untreated SMA patient-derived primary fibroblasts (U) and normalized SMN protein after *in vitro* treatment with risdiplam (R, light blue) or nusinersen (N, coral). Data are represented as the average of the technical triplicate ± standard deviation. Regression line, Pearson correlation coefficient (R), *P*-value (p) and the coefficient of determination (R^2) are displayed. U = untreated; N = nusinersen; R = risdiplam; a.u. = arbitrary unit; CN = copy number.

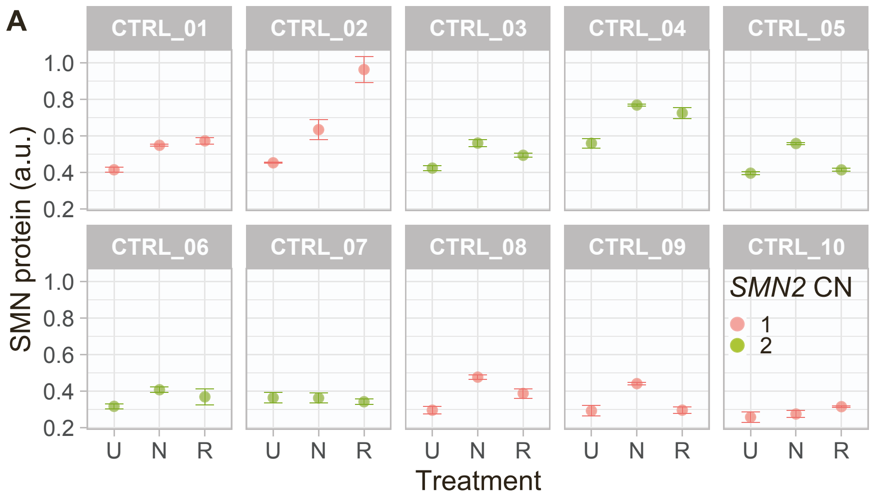

Figure S7. Treatment effect across control cell lines.

(A) Normalized SMN protein expression levels in control patient-derived primary fibroblasts untreated (U) and after *in vitro* treatment with either nusinersen (N) or risdiplam (R). Data are represented as the average of the technical triplicate ± standard deviation. U = untreated; N = nusinersen; R = risdiplam; a.u. = arbitrary unit; CN = copy number.

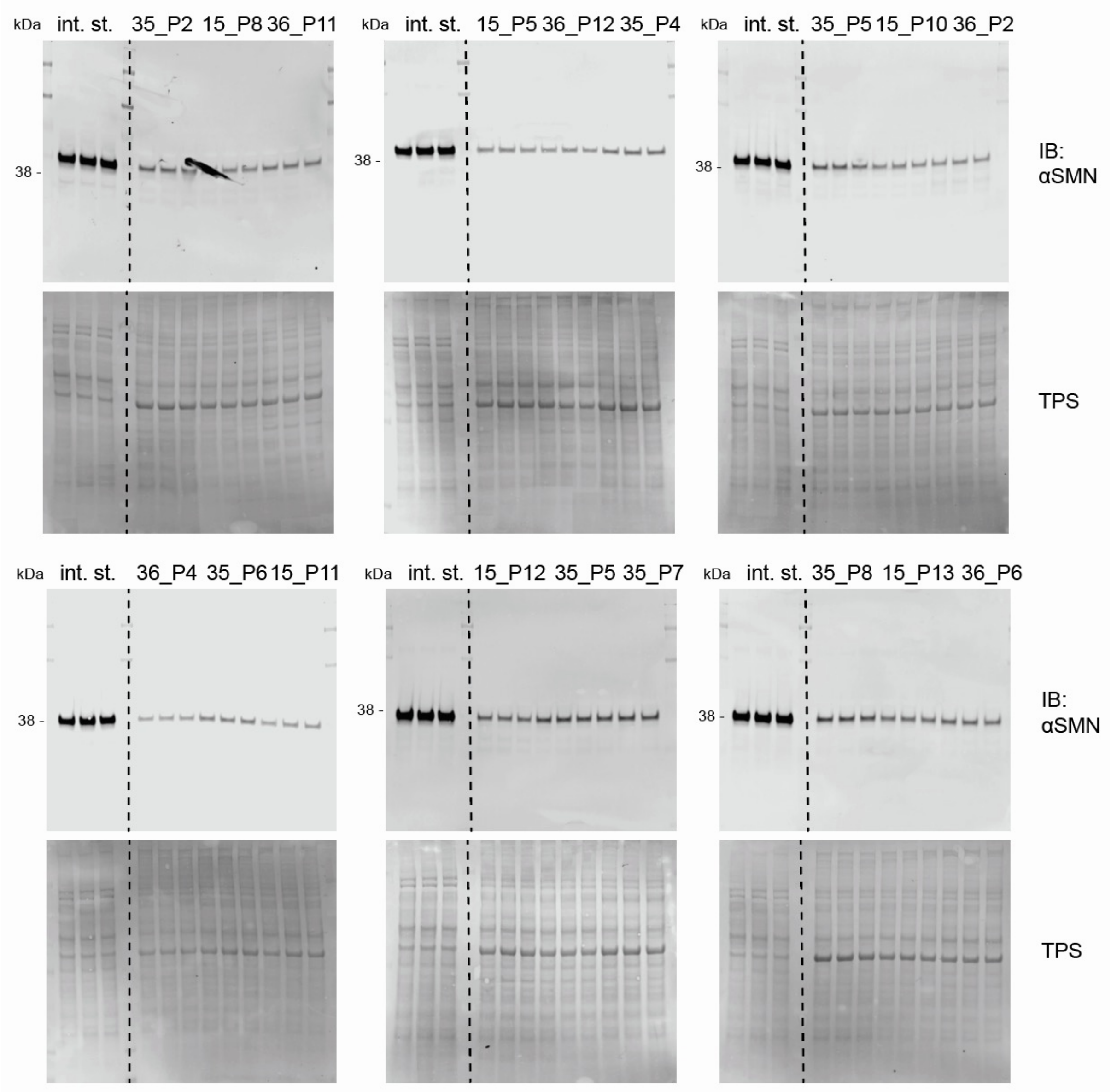

Figure S8A. Uncropped SMN western blots for Fig. S2 (SMN at different passage numbers). TPS = total protein staining; in. st = internal standard.

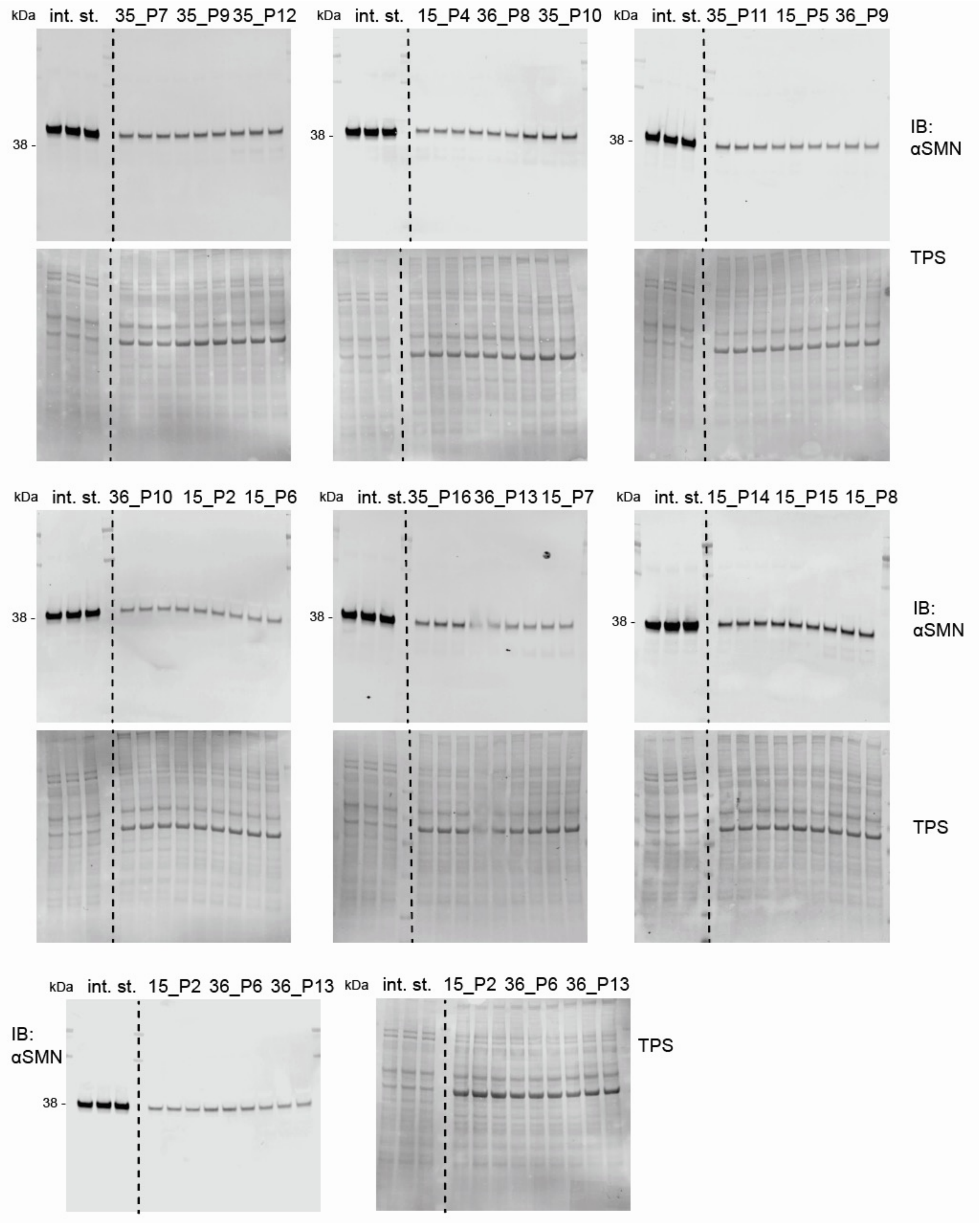

Figure S8B. Uncropped SMN western blots for Fig. S2 (SMN at different passage numbers). TPS = total protein staining; in. st = internal standard.

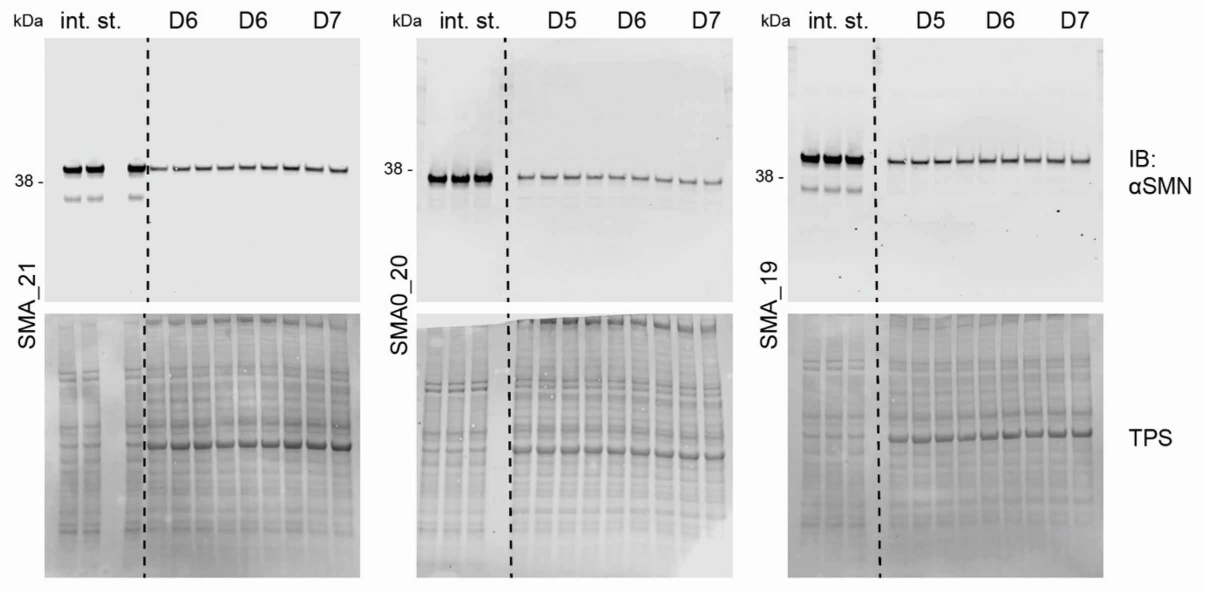

Figure S8C. Uncropped SMN western blots for Fig. S2 (SMN at different confluences and days in culture). TPS = total protein staining; in. st = internal standard.

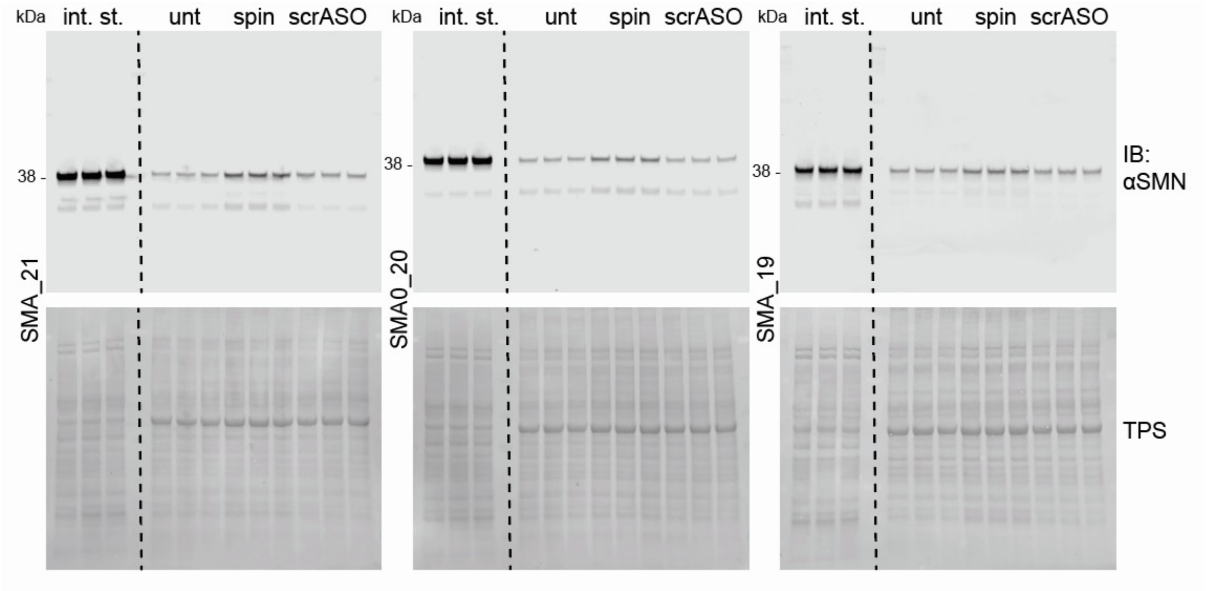

Figure S8D. Uncropped SMN western blots for Fig. S2. SMN protein levels in patient-derived fibroblasts untreated (U), treated with nusinersen (N) and treated with scrASO (scrASO). TPS = total protein staining; in. st = internal standard.

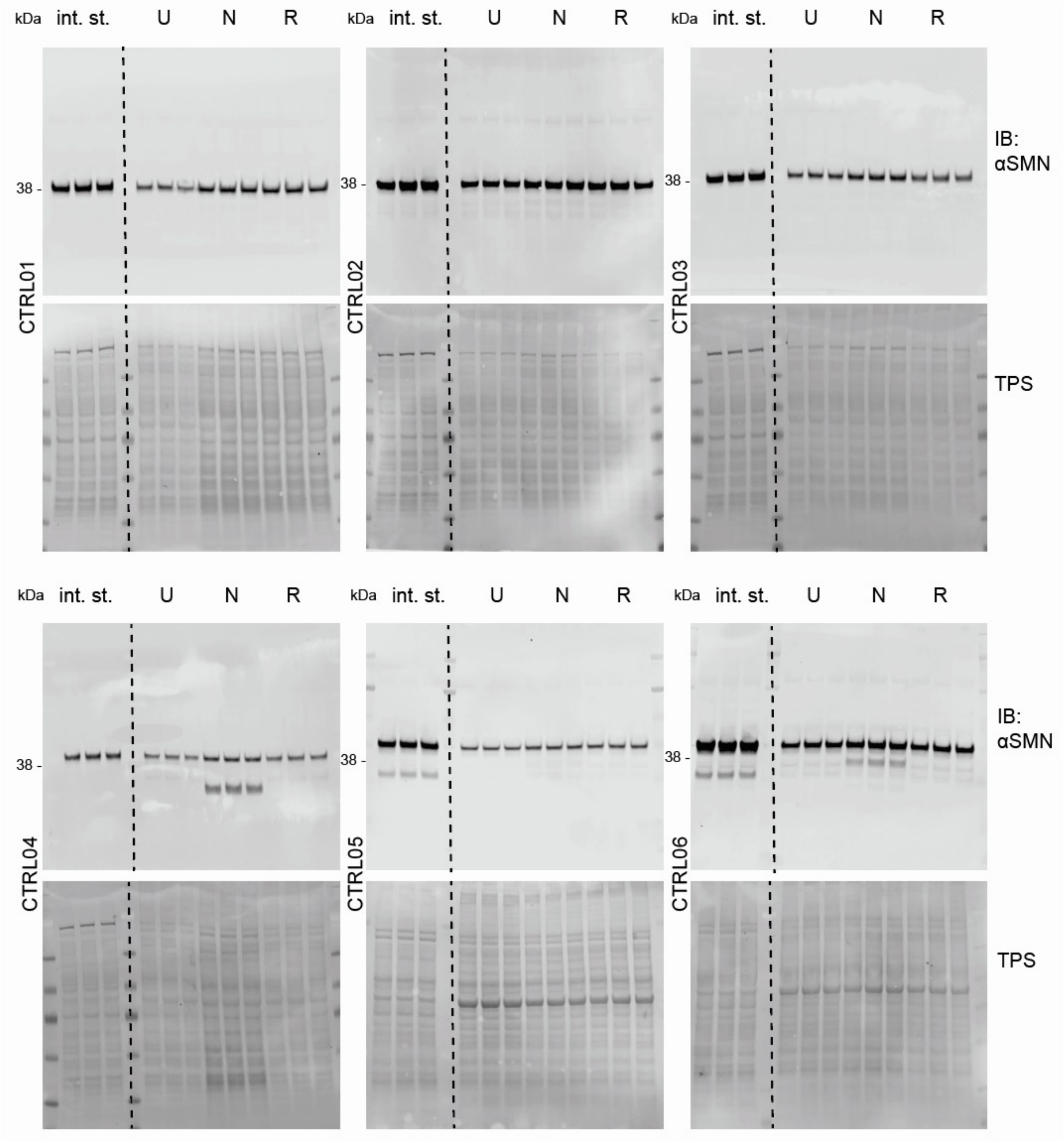

Figure S8E. Uncropped SMN western blots for Fig. 1, 2, 4, S5, S6, S7. SMN protein levels in patient-derived fibroblasts untreated (U), treated with nusinersen (N) and treated with risdiplam (R). TPS = total protein staining; in. st = internal standard.

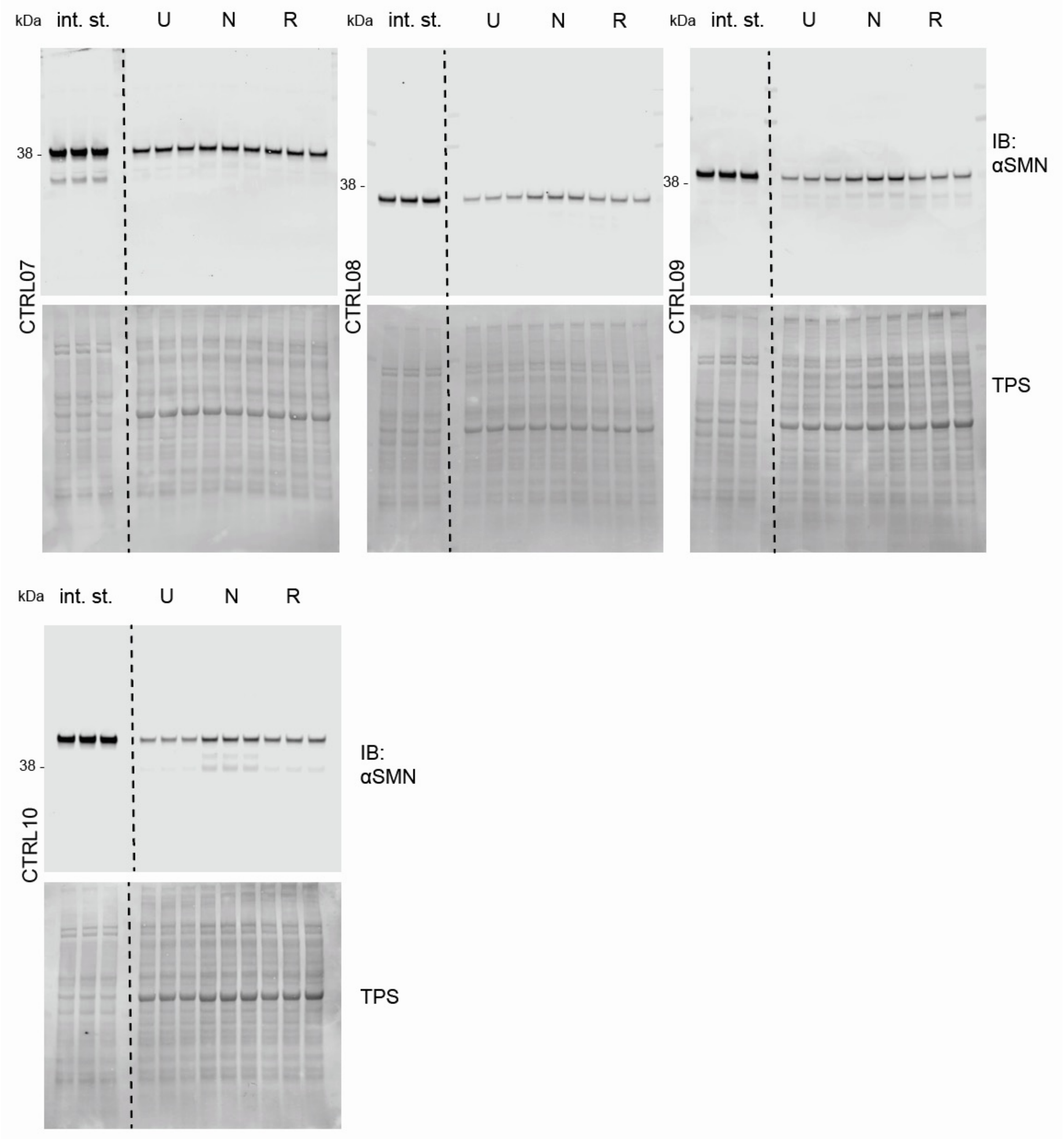

Figure S8F. Uncropped SMN western blots for Fig. 1, 2, 4, S5, S6, S7. SMN protein levels in patient-derived fibroblasts untreated (U), treated with nusinersen (N) and treated with risdiplam (R). TPS = total protein staining; in. st = internal standard.

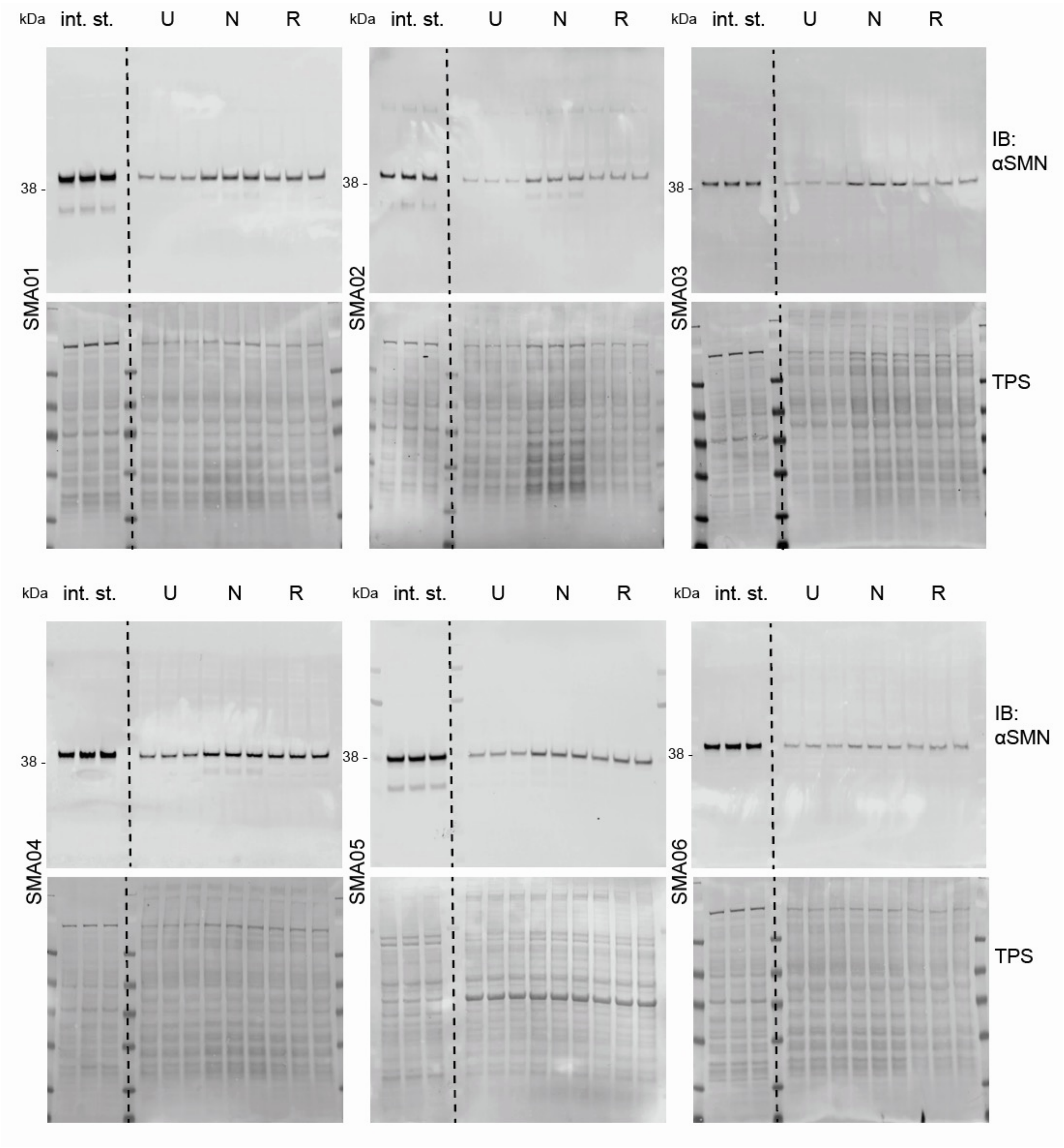

Figure S8G. Uncropped SMN western blots for Fig. 1, 2, 3, 4, S5, S6. SMN protein levels in patient-derived fibroblasts untreated (U), treated with nusinersen (N) and treated with risdiplam (R). TPS = total protein staining; in. st = internal standard.

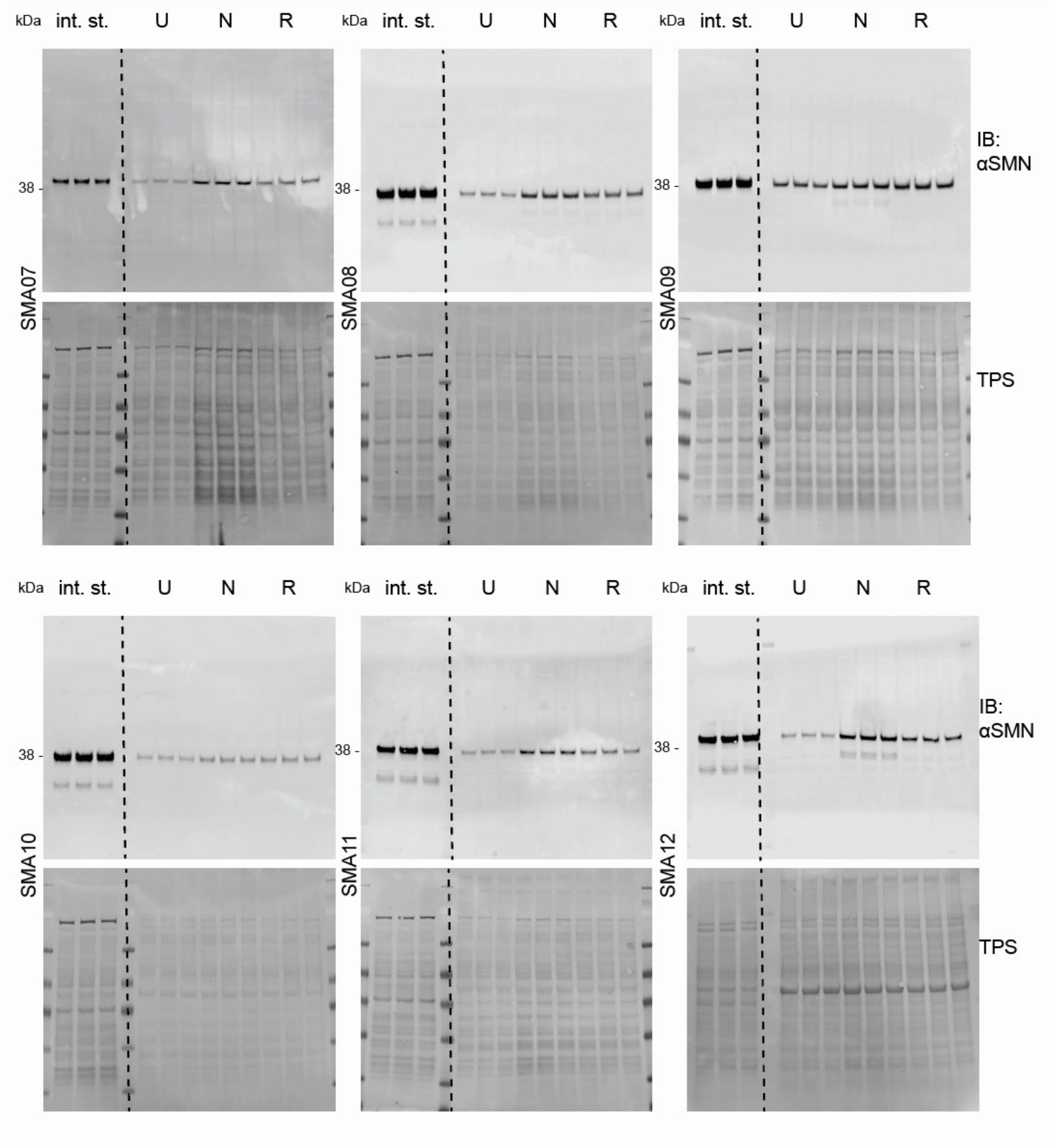

Figure S8H. Uncropped SMN western blots for Fig. 1, 2, 3, 4, S5, S6. SMN protein levels in patient-derived fibroblasts untreated (U), treated with nusinersen (N) and treated with risdiplam (R). TPS = total protein staining; in. st = internal standard.

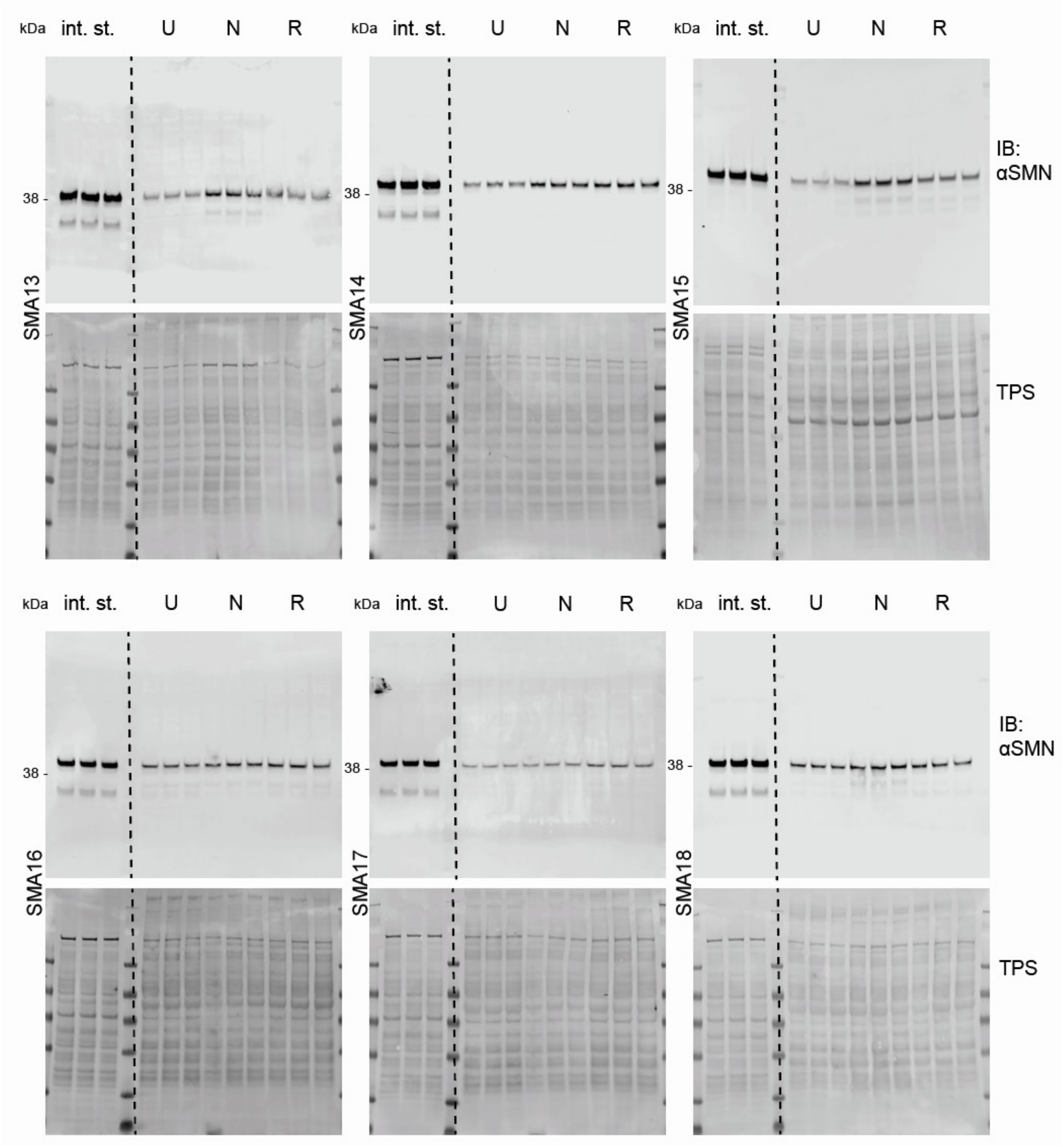

Figure S8I. Uncropped SMN western blots for Fig. 1, 2, 3, 4, S5, S6. SMN protein levels in patient-derived fibroblasts untreated (U), treated with nusinersen (N) and treated with risdiplam (R). TPS = total protein staining; in. st = internal standard.

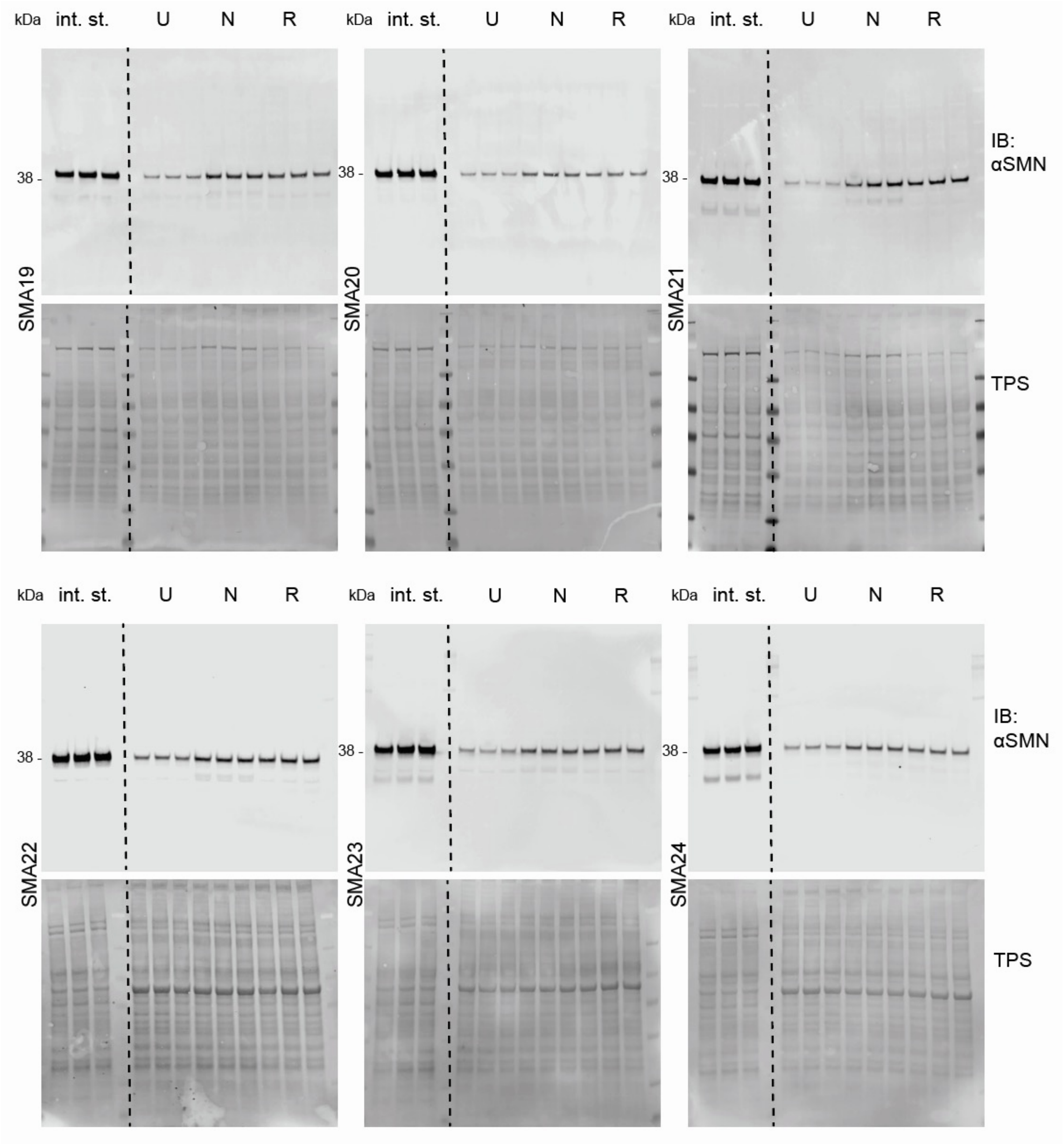

Figure S8J. Uncropped SMN western blots for Fig. 1, 2, 3, 4, S5, S6. SMN protein levels in patient-derived fibroblasts untreated (U), treated with nusinersen (N) and treated with risdiplam (R). TPS = total protein staining; in. st = internal standard.

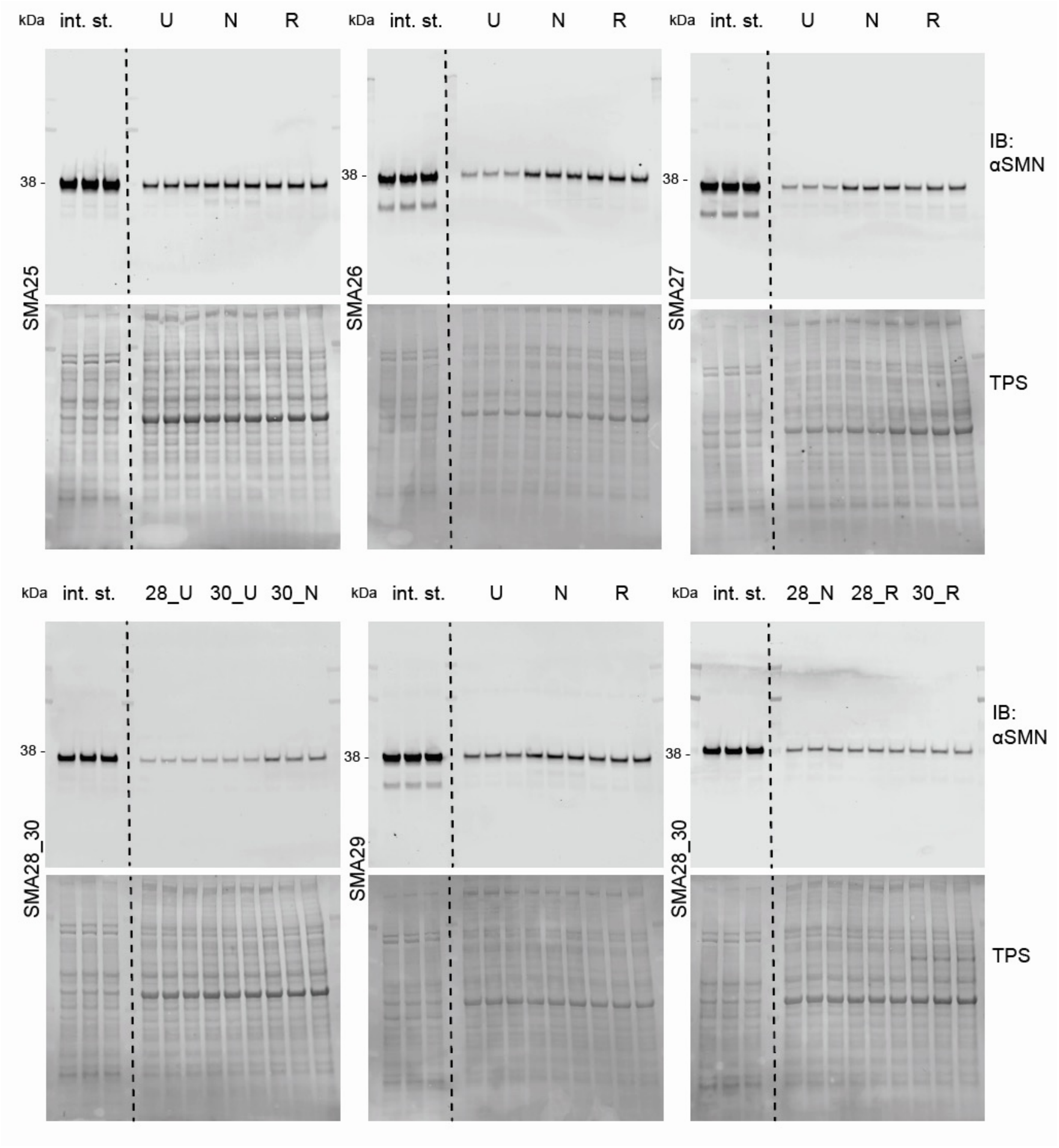

Figure S8K. Uncropped SMN western blots for Fig. 1, 2, 3, 4, S5, S6. SMN protein levels in patient-derived fibroblasts untreated (U), treated with nusinersen (N) and treated with risdiplam (R). TPS = total protein staining; in. st = internal standard.

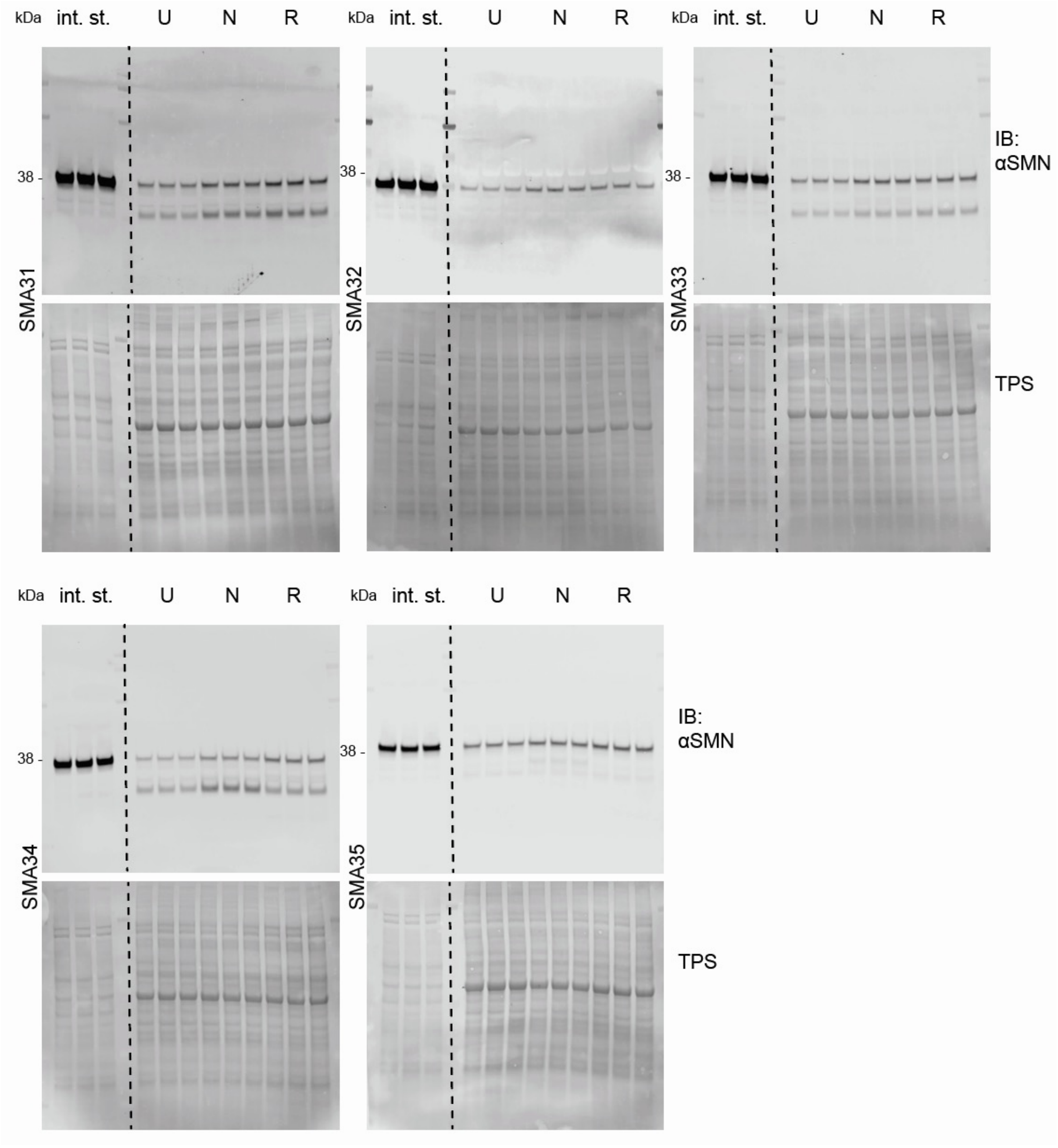

Figure S8L. Uncropped SMN western blots for Fig. 1, 2, 3, 4, S5, S6. SMN protein levels in patient-derived fibroblasts untreated (U), treated with nusinersen (N) and treated with risdiplam (R). TPS = total protein staining; in. st = internal standard.
